# Genome Editing of *Babesia bovis* Using the CRISPR/Cas9 System

**DOI:** 10.1128/mSphere.00109-19

**Published:** 2019-06-12

**Authors:** Hassan Hakimi, Takahiro Ishizaki, Yuto Kegawa, Osamu Kaneko, Shin-ichiro Kawazu, Masahito Asada

**Affiliations:** a Department of Protozoology, Institute of Tropical Medicine (NEKKEN), Nagasaki University, Nagasaki, Japan; b Program for Nurturing Global Leaders in Tropical and Emerging Communicable Diseases, Graduate School of Biomedical Sciences, Nagasaki University, Nagasaki, Japan; c National Research Center for Protozoan Diseases, Obihiro University of Agriculture and Veterinary Medicine, Obihiro, Hokkaido, Japan; University at Buffalo

**Keywords:** *Babesia bovis*, CRISPR/Cas9, genome editing, thioredoxin peroxidase

## Abstract

Babesia bovis, the most virulent causative agent of bovine babesiosis, is prevalent in tropical and subtropical regions of the world. Although the whole-genome sequence was released more than a decade ago, functional analysis of the genomics of this parasite is hampered by the limited breadth of genetic engineering tools. In this study, we implemented the clustered regularly interspaced short palindromic repeat (CRISPR)/Cas9 system for B. bovis and demonstrated its potential for genome editing. Cas9 and human dihydrofolate reductase (hDHFR) were simultaneously expressed by the B. bovis *elongation factor-1α* bidirectional promoter, and a single guide RNA was expressed via the B. bovis *U6 spliceosomal RNA* promoter. Using a single plasmid construct, we were able to add an epitope tag to spherical body protein 3 (SBP3), introduce a point mutation into *thioredoxin peroxidase 1* (*tpx-1*) to impair the function of the product, and replace the *tpx-1* open reading frame with the other protein. Epitope tagging of SBP3 was efficient using this system, with a negligible number of remaining wild-type parasites and a pure transgenic population produced by allelic replacement of *tpx-1*. This advancement in genetic engineering tools for B. bovis will aid functional analysis of the genome and underpin characterization of candidate drug and vaccine targets.

**IMPORTANCE** Babesia bovis is the most virulent cause of bovine babesiosis worldwide. The disease consequences are death, abortion, and economical loss due to reduced milk and meat production. Available vaccines are not effective, treatment options are limited, and emergence of drug and acaricide resistance has been reported from different regions. There is an urgent need to identify new drug and vaccine targets. Greater than half of the genes in B. bovis genome, including several expanded gene families which are unique for *Babesia* spp., have no predicted function. The available genetic engineering tools are based on conventional homologous recombination, which is time-consuming and inefficient. In this study, we adapted the CRISPR/Cas9 system as a robust genetic engineering tool for B. bovis. This advancement will aid future functional studies of uncharacterized genes.

## Abstract (precis)

Babesia bovis is the most virulent cause of bovine babesiosis worldwide. The disease consequences are death, abortion, and economical loss due to reduced milk and meat production. Available vaccines are not effective, treatment options are limited, and emergence of drug and acaricide resistance has been reported from different regions. There is an urgent need to identify new drug and vaccine targets. Greater than half of the genes in B. bovis genome, including several expanded gene families which are unique for *Babesia* spp., have no predicted function. The available genetic engineering tools are based on conventional homologous recombination, which is time-consuming and inefficient. In this study, we adapted the CRISPR/Cas9 system as a robust genetic engineering tool for B. bovis. This advancement will aid future functional studies of uncharacterized genes.

## INTRODUCTION

Babesia bovis is the most virulent *Babesia* parasite that infects cattle and produces severe clinical symptoms and occasional death in naive animals. The lack of an effective vaccine and the emergence of drug and acaricide resistance in the field are the main challenges to control of bovine babesiosis (1). Following the completion of genome sequences for B. bovis and several other *Babesia* spp. (2–4), classical genetic modification techniques for these parasites have been established (5–9). These methods are based on homologous recombination, which introduces plasmid DNA containing a drug marker into the target locus followed by selection of these transgenic parasites by a specific drug. However, these methods have several limitations; namely, the efficiency of the genome integration of the plasmid is low, drug selection process is time-consuming, and, more importantly, the insertion of the selection marker into the genome is not ideal for some phenotypic assays and also restricts the manipulation of multiple gene loci in a single parasite.

Recently, precise and site-specific genome editing technologies such as zinc finger nuclease (ZFN), transcription activator-like effector nuclease (TALEN), and CRISPR/Cas9 (clustered regularly interspaced short palindromic repeat and Cas9 endonuclease-mediated genome editing) technologies have been developed. In these methods, a nuclease produces a double-strand break (DSB) in the target region which is repaired by homologous recombination in the presence of a template DNA fragment or through nonhomologous end joining. This process can produce errors such as deletions or insertions in the DSB site. ZFN was successfully adapted for genome editing of Plasmodium falciparum and P. vivax, two agents causing human malaria (10, 11). Unlike ZFN and TALEN, for which significant effort is needed for the design and production of expensive nucleases, the CRISPR/Cas9 system needs only a specific single guide RNA (sgRNA) to direct and guide Cas9 nuclease to the target site (12). The CRISPR/Cas9 system is derived from a prokaryotic adaptive immune mechanism against invading viruses and plasmids that produce a DSB at a specific site of DNA (12). CRISPR/Cas9 genome editing has been adapted for several protozoan parasites, including *Plasmodium* and *Toxoplasma* (13–15), and has been used for functional analysis of several genes in these parasites (16).

Nonhomologous end joining machinery does not exist in *Babesia* spp.; therefore, classical genetic manipulation technologies based on homologous recombination have been used for gene functional studies in B. bovis during the erythrocytic and tick stages (6, 17–19). Functional studies of this parasite have been hampered by the limited breadth of available tools and inefficiency of transfection. In this study, we designed a basic plasmid expressing Cas9 and human dihydrofolate reductase (hDHFR) for B. bovis and evaluated its usefulness in this pathogen by examining the efficiency of epitope tagging, introduction of a point mutation, and gene replacement.

## RESULTS

### Addition of a myc tag epitope at the C-terminal end of B. bovis SBP3.

**FIG 1:**
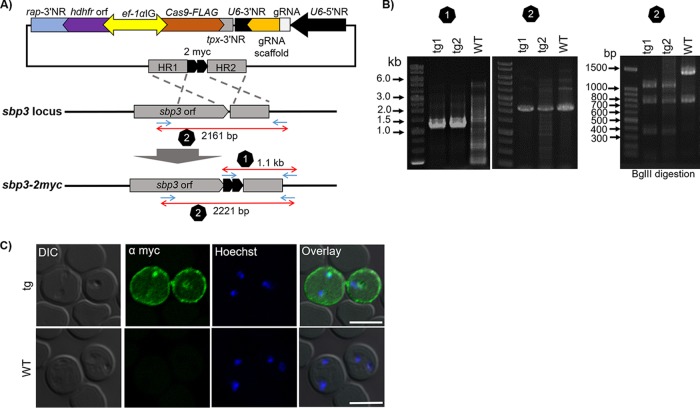
CRISPR/Cas9-mediated C-terminal epitope tagging of B. bovis SBP3. (A) Schematic of BbU6-Cas9-hDHFR-sbp3-myc. *rap*-3′NR, *rhoptry associated protein* 3′ noncoding region; *hdhfr* orf, *human dihydrofolate reductase* open reading frame; *ef-1a*IG, *elongation factor-1α* intergenic region; *tpx*-3′NR, *thioredoxin peroxidase-1* 3′ noncoding region; U6-3′NR, *U6 spliceosomal RNA* 3′ noncoding region; gRNA, guide RNA; HR, homologous region. (B) Agarose gel electrophoresis separation of products following the diagnostic PCR to confirm the integration of the sequence encoding myc epitopes. (C) Indirect immunofluorescence microscopy of transgenic B. bovis having SBP3 tagged with myc epitopes and WT (α-myc, green). The smear was prepared from the parasites that appeared 10 days after drug selection. The parasite nuclei were stained with Hoechst 33342 (Hoechst, blue). Scale bar = 5 μm. DIC, differential interference contrast.

To adapt the CRISPR/Cas9 system to B. bovis, we constructed a plasmid (BbU6-Cas9-hDHFR-sbp3-myc) to insert a sequence encoding a 2-myc tag epitope at the 3′ end of the B. bovis *sbp3* open reading frame (orf; Fig. 1A). In this plasmid, expression of Cas9 and expression of hDHFR were driven simultaneously by *ef-1α* intergenic region (*ef-1α* IG), which works as a bidirectional promoter. The B. bovis *U6 spliceosomal RNA* promoter drove the expression of the sgRNA, which contained a 20-nucleotide guide RNA that targets a region at the 3′ end of B. bovis *sbp3*. We chose SBP3 since it is expressed in the parasite-infected red blood cell (iRBC) cytoplasm and exhibits a characteristic staining pattern (20). The iRBCs were transfected with a single circular plasmid, and parasites appeared 10 days after WR99210 selection. The sgRNA transcripts were detected initially by reverse transcriptase PCR (RT-PCR), verifying the B. bovis *U6 spliceosomal RNA* promoter activity (see Fig. S1A and B in the supplemental material). An indirect immunofluorescence antibody test (IFAT) using anti-FLAG antibody confirmed the expression of FLAG-tagged Cas9 in the parasite nucleus (Fig. S1C). The correct insertion of the sequence for the myc tag was evaluated by PCR, which amplified 1.1-kb DNA fragments (Fig. 1A and B). To confirm the absence of the remaining wild-type (WT) parasites, PCR-restriction fragment length polymorphism (PCR-RFLP) analysis was performed using primers out of recombination sites, which produced 2,161-bp and 2,221-bp fragments for the WT and myc-tagged parasites, respectively. The PCR products were digested with BglII, which produced 770- and 1,391-bp DNA fragments for the WT strain and 30-, 357-, 770-, and 1,064-bp DNA fragments for myc-tagged parasites. This PCR-RFLP analysis confirmed the absence of the remaining WT parasites in the transgenic populations (Fig. 1A and B). IFAT was performed using anti-myc antibody to check if the myc tag was fused to SBP3, and patchy staining within the iRBC cytoplasm and iRBC periphery was seen (Fig. 1C), in agreement with a report showing the export of SBP3 into the iRBC and its association with the iRBC membrane (20). Survey of 100 parasites revealed all to be fluorescence positive, indicating efficient tagging and negligible remaining numbers of WT parasites.

10.1128/mSphere.00109-19.1 FIG S1 Expression of Cas9 and sgRNA in B. bovis from a single plasmid. (A) Schematic of the plasmid construct for coexpression of Cas9 and hDHFR using the B. bovis *ef1-α* intergenic region and sgRNA under the control of a B. bovis *U6 spliceosomal RNA* promoter. For abbreviations, see the Fig. 1 legend. (B) RT-PCR to confirm the expression of sgRNA. PCR was done using cDNA after reverse transcription (RT+) or using RNA without reverse transcription (RT-) as a template. (C) Indirect immunofluorescence microscopy of transgenic B. bovis and WT strains using anti-FLAG antibody (α-FLAG, green) to detect FLAG-tagged Cas9. The smear was prepared from the parasites that appeared 10 days after drug selection. The parasite nuclei were stained with Hoechst 33342 (Hoechst, blue). Scale bar = 5 μm. Download FIG S1, TIF file, 0.6 MB. Copyright © 2019 Hakimi et al. 2019 Hakimi et al. This content is distributed under the terms of the Creative Commons Attribution 4.0 International license.

### Introduction of a point mutation into B. bovis *tpx-1* that impairs its function.

**FIG 2:**
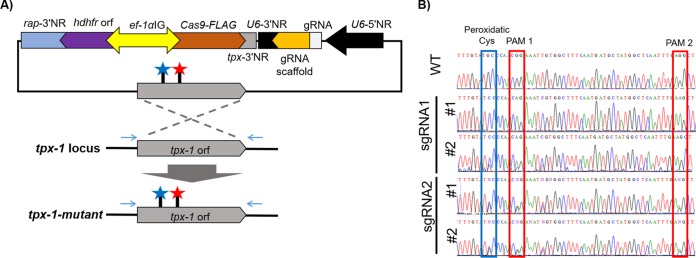
CRISPR/Cas9-mediated nucleotide editing of B. bovis *tpx-1*. (A) Diagram illustrating the plasmid design and the strategy to produce two point mutations at peroxidatic Cys and protospacer-adjacent motif (PAM) sites. The primer sites for sequencing are indicated as arrows out of the recombination site. For abbreviations, see the Fig. 1 legend. (B) Electrograms of the nucleotide sequence covering the modified sites for peroxidatic Cys and PAM in the wild-type (WT) and transfected parasite populations.

Tpx-1 is a cytoplasmic antioxidant enzyme peroxiredoxin with an essential peroxidatic Cys at its catalytic site functioning to reduce the peroxide substrate (21–23). To evaluate the CRISPR/Cas9 system for making a point mutation in a target gene, without integrating a selectable marker, we made the BbU6-Cas9-hDHFR-tpx-1-mutant plasmid with donor DNA from a *tpx-1* orf containing a mutation to change the peroxidatic Cys (Cys_49_) to Ser (Fig. 2A). The *tpx-1* orf was amplified following the appearance of transgenic parasites, and sequencing confirmed the presence of the desired mutation and the protospacer-adjacent motif (PAM) sequence (Fig. 2B). Transfectants from 2 different sgRNA showed efficacies ranging from a mixture of WT and mutants to a pure mutant population with negligible remaining levels of WT locus, indicating various efficiencies of sgRNA in guiding Cas9 into the target locus (Fig. 2B).

### Deletion of B. bovis *tpx-1* gene locus and replacement with green fluorescent protein (GFP).

**FIG 3:**
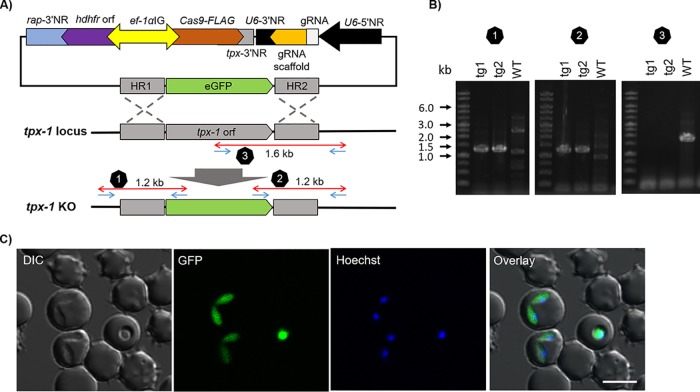
CRISPR/Cas9-mediated replacement of B. bovis *tpx-1* with *gfp*. (A) Schematic showing the replacement of the B. bovis *tpx-1* orf with the *gfp* orf. For abbreviations, see Fig. 1 legend. (B) PCR to confirm the recombination event. PCR 1 primer pair, BbTpx-1-5NR-IntegF and eGFP-R; PCR 2 primer pair, eGFP-F and BbTpx-1-3NR-IntegR; PCR3 primer pair, BbTPx1-F and BbTpx-1-3NR-IntegR. (C) Live fluorescence microscopy images of GFP-expressing parasites. The parasite nuclei were stained with Hoechst 33342 (Hoechst, blue). Scale bar = 5 μm.

To validate the CRISPR/Cas9 system for making a gene knockout (KO) in B. bovis, we targeted *tpx-1*, which is not essential for the erythrocytic stage (6). A single plasmid, BbU6-Cas9-hDHFR-tpx-1KO-GFP, was designed to replace the *tpx-1* orf with the *gfp* orf (Fig. 3A). The diagnostic PCR performed with primer pairs to amplify the 5′ side or 3′ side of the modified gene locus successfully amplified 1.2-kb DNA fragments from transgenic parasites (Fig. 3B, panels 1 and 2). The presence or absence of the *tpx-1* WT locus was evaluated by PCR-3, showing 1.6-kb DNA fragments for the WT parasites and no amplified product for the transgenic parasites (Fig. 3B, panel 3), indicating a pure KO parasite population. To evaluate whether the transfected plasmid was episomally maintained or integrated into the genome, we performed Southern blot analysis (Fig. S2). A probe targeting *gfp* orf detected two bands, namely, a 5.8-kb band as expected from the modified *tpx-1* locus and a 7.8-kb band which was similar in size to the control plasmid. To further examine whether the 7.8-kb band was derived from the episomal or from the genome-integrated plasmid, additional Southern blotting was done using AatII, which cuts the *tpx-1* locus but not CRISPR/Cas9 plasmid. The expected band size for the episomal plasmid was 4.5 kb; however, both transgenic parasites showed a higher band, suggesting integration of the plasmid into the genome. Additionally, a probe targeting *cas9* orf showed a single 8.6-kb band similar to that seen with the control plasmid. Altogether, Southern blot analysis results indicated integration of the CRISPR/Cas9 plasmid into the genome. Live fluorescence microscopy of over 100 parasites showed GFP expression from all parasites, representing good agreement with our PCR result of having a pure KO population.

10.1128/mSphere.00109-19.2 FIG S2 Schematic diagram and Southern blot analysis of *tpx-1* KO locus. Genomic DNA from two *tpx-1* KO clones and from WT and plasmid constructs was digested with HindIII, AatII, or BamHI and SalI and hybridized with GFP or Cas9 probes. Download FIG S2, TIF file, 0.3 MB. Copyright © 2019 Hakimi et al. 2019 Hakimi et al. This content is distributed under the terms of the Creative Commons Attribution 4.0 International license.

### B. bovis *tpx-1* KO and mutant parasites are sensitive to sodium nitroprusside.

**FIG 4:**
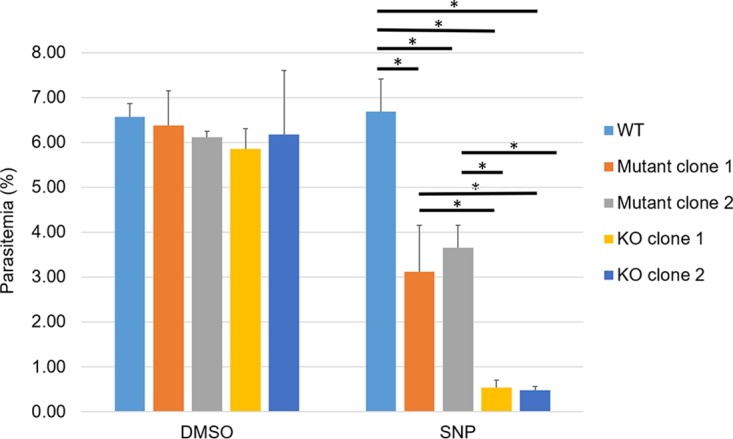
Sensitivity of B. bovis wild-type (WT), *tpx-1* knockout (KO), and *tpx-1* mutant (Mutant) parasites to sodium nitroprusside (SNP). Two clones of the B. bovis *tpx-1* KO strain, two clones of a *tpx-1* mutant for which the peroxidatic Cys was replaced with Ser, and the parental WT parasites were incubated with 10 μM SNP in the culture medium for 4 days. The level of parasitemia on day 4 is plotted with means ± standard deviations (SD) from the triplicated well cultures (*, *P* < 0.0001 [one-way ANOVA followed by Tukey’s multiple-comparison test]).

To determine whether the *tpx-1* KO and mutant parasite lines generated by the CRISPR/Cas9 system in this study displayed altered phenotypes, we took advantage of a report showing that B. bovis *tpx-1* KO parasites were more sensitive to sodium nitroprusside (SNP) than WT parasites (17). Two KO clones and two mutant clones for which the peroxidatic Cys was changed to Ser were cultured together with WT parental parasites in the presence of 10 μM SNP. A 4-day exposure to SNP resulted in a significant decrease in the growth rate of the *tpx-1* KO and mutant clones compared to that of WT (*P* < 0.0001, Fig. 4). In addition, the levels of parasitemia seen with the KO clones were significantly lower than those seen with the mutant clones, indicating that the KO clones were more sensitive to SNP than the mutant lines (*P* < 0.0001). No significant change was seen in the growth rates of negative-control parasites cultured with dimethyl sulfoxide (DMSO).

## DISCUSSION

Existing tools for genetic engineering of B. bovis are based on classical homologous recombination driven by insertion of a selection marker into the genome of the parasite. The limited availability of selection markers limits the number of genes that can be targeted in a single parasite. Genome modification using the CRISPR/Cas9 system does not require the insertion of a selection marker into the genome, thus allowing the possibility of targeting an unlimited number of genes in a single parasite. Disruption of multiple genes is important in functional studies of multigene families or genes with redundant functions. Here, we report the usage of the CRISPR/Cas9 system for B. bovis, which permitted, using a single plasmid, epitope tagging or the introduction of a point mutation and gene replacement. Two independent replacements of *tpx-1* to *gfp* using two different sgRNAs produced pure transgenic parasite populations and no detectable WT parasites. Epitope tagging of SBP3 was also very efficient, with negligible levels of WT parasites remaining. However, introduction of a point mutation in *tpx-1* using 2 different sgRNAs showed different percentages of transgenic parasites, indicating that the choice of sgRNA determined the efficacy of Cas9 targeting. These results demonstrate the utility and potential of the plasmid designed in this study. We showed that genome editing could be achieved efficiently; however, the plasmid tended to be integrated into the genome which could be removed by using a negative-selection marker (24). It was reported previously that the CRISPR/Cas9-based genome editing method using one plasmid design resulted in the integration of the plasmid into the genome in the rodent malaria parasite Plasmodium yoelii (25, 26). A novel ribosome-mediated CRISPR system was recently reported that allows expression of several sgRNAs, prevents integration of the plasmid into the genome, and produces a marker-free P. yoelii strain (26).

Tpx-1 has two conserved Cys residues; one is a peroxidatic Cys (catalysis site) and the other a resolving Cys making a disulfide bond with the peroxidatic Cys following catalysis (27). In a published study, the deletion of B. bovis Tpx-1 increased parasite sensitivity to nitrosative stress (17); however, the importance of the peroxidatic Cys was not assessed. In this study, we mutated the peroxidatic Cys to Ser and found that this mutation had less impact on the sensitivity to reactive nitrogen species (RNS) than the complete deletion of the *tpx-1* orf. This indicates the contribution of other factors, such as a resolving Cys, in the antioxidant activity of this enzyme. In P. falciparum, Tpx-1 was shown to reduce peroxinitrite, the major SNP-derived RNS, with an unidentified catalytic center (28). Here we showed that other catalytic sites in addition to peroxidatic Cys exist to react with and detoxify RNS.

This is the first demonstration of CRISPR/Cas9 in *Babesia* parasites. We showed the potential of CRISPR/Cas9 in editing 2 different genes in B. bovis; however, implementation of this system for diverse genetic loci as well as parallel comparisons with a conventional homologous recombination system may give more insights into the efficiency of CRISPR/Cas9 in B. bovis. Implementation of inducible Cas9 or Cas13 targeting RNA will further develop the capabilities of this tool kit for use in analysis of the functional genomics of B. bovis (19). Considering the limited number of selectable markers for B. bovis, the production of marker-free parasites is invaluable for performing consecutive genome manipulations.

## MATERIALS AND METHODS

### Parasite culture.

B. bovis strain Texas was maintained *in vitro* with purified bovine RBCs (Nippon Bio-Supply Center, Tokyo, Japan) and GIT medium (Wako Pure Chemical Industries, Osaka, Japan) using a microaerophilic stationary-phase culture system.

### Plasmid constructs.

A schematic of the Cas9-expressing plasmid is shown in Fig. S1 in the supplemental material. The primers used for construction of the plasmid are listed in Table S1 in the supplemental material. A DNA fragment encoding N-terminally FLAG-tagged Cas9 was amplified from pDC2-cam-Cas9-U6-hDHFR (GenBank accession number KY574493). A DNA fragment containing *hdhfr* and B. bovis *rhoptry associated protein* 3′ noncoding region (*rap* 3′NR) was amplified from a B. bovis GFP-expressing plasmid (6). B. bovis *elongation factor-1α* intergenic region (*ef-1α* IG) and *thioredoxin peroxidase-1* (*tpx-1*) 3′NR were PCR amplified from B. bovis genomic DNA (gDNA). DNA fragments for Cas9 and *ef-1α* IG were cloned into the EcoRI site of pBluescript SK using an In-Fusion HD cloning kit (TaKaRa Bio Inc., Otsu, Japan). *hdhfr-rap* 3′NR and *tpx-1* 3′NR were cloned into SmaI and HindIII sites, respectively, to make the BbCas9-hDHFR plasmid. B. bovis U6 spliceosomal RNA gene was found by a homology search in PiroplasmaDB using as a query the P. falciparum U6 spliceosomal RNA (PF3D7_1341100). B. bovis U6 spliceosomal RNA gene was not annotated in PiroplasmaDB and located on chromosome 3 between BBOV_III004870 and BBOV_III004880. A total of 600 bp of the 5′NR (U6 5′NR) and 100 bp of the 3′NR (U6 3′NR) were PCR amplified from B. bovis gDNA and used as a promoter to drive sgRNA and a terminator, respectively. gRNA scaffold was amplified from pDC2-cam-Cas9-U6-hDHFR. B. bovis U6 5′NR, gRNA scaffold, and U6 3′NR were cloned into the EcoRI site of pBluescript SK to make BbU6-gRNA. The final Cas9-expressing plasmid (BbU6-Cas9-hDHFR) was constructed by inserting BbCas9-hDHFR and BbU6-gRNA into the EcoRI site of pBluescript SK. The target sgRNA was inserted into the AarI site in BbU6-Cas9-hDHFR using T4 DNA ligase (New England Biolabs, Beverly, MA, USA). Insertion of homologous regions (HRs) for epitope tagging, introduction of a point mutation, and gene replacement were performed using PCR amplification with specific primers (Table S1), and the resulting sequences were inserted into BamHI site of BbU6-Cas9-hDHFR using an In-Fusion HD cloning kit. All plasmids were purified with a Qiagen Plasmid Maxi kit (Qiagen, MD, USA) following the manufacturer’s instructions, and the inserted DNA sequences were confirmed by sequencing.

10.1128/mSphere.00109-19.3 TABLE S1 List of primers used in this study. Download Table S1, XLSX file, 0.01 MB. Copyright © 2019 Hakimi et al. 2019 Hakimi et al. This content is distributed under the terms of the Creative Commons Attribution 4.0 International license.

### Transfection of parasites.

The preparation and transfection of B. bovis were performed as previously described (6). Briefly, B. bovis iRBCs were washed twice with phosphate-buffered saline (PBS) and once with cytomix buffer (120 mM KCl, 0.15 mM CaCl_2_, 10 mM K_2_HPO_4_, 10 mM KH_2_PO_4_, 25 mM HEPES, 2 mM EGTA, 5 mM MgCl_2_, 100 μg/ml bovine serum albumin, 1 mM hypoxanthine; pH 7.6). A 10-μg volume of the circular plasmid construct in 10 μl of cytomix buffer was mixed with 90 μl of Amaxa Nucleofector human T-cell solution and then combined with 100 μl of washed packed iRBCs. Transfection was done using a Nucleofector device (program v-024; Amaxa Biosystems, Cologne, Germany), and iRBCs were immediately transferred into 1 ml of culture containing 10% bovine RBCs. WR99210 (10 nM) was added 1 day after the transfection to episomally maintain the plasmid and produce a DSB.

### Tagging of B. bovis spherical body protein 3 (SBP3) with 2-myc epitopes.

To test the transfection efficiency with BbU6-Cas9-hDHFR, we transfected B. bovis iRBCs with a plasmid containing HRs and a sequence encoding a 2-myc epitope to tag the C-terminal end of B. bovis SBP3 (BbU6-Cas9-hDHFR-sbp3-myc). After the appearance of parasites following drug selection, DNA was extracted and diagnostic PCR was done using Myc-F and BbSBP3-3rec-R primers. To differentiate WT and myc-tagged parasites, PCR-RFLP analysis was done using BbSBP3-5GI-F and BbSBP3-3GI-R primers followed by BglII digestion. RNA was additionally extracted from transgenic and WT parasites to confirm the expression of chimeric sgRNA. Primer pairs of BbSBP3-gRNA-1F or BbSBP3-gRNA-2F and gRNA-scaffold-R-IF were used to amplify a 100-bp DNA fragment in a reverse transcriptase PCR (RT-PCR).

### Deletion of B. bovis *tpx-1*and replacement with GFP.

BbU6-Cas9-hDHFR-tpx-1KO-GFP was designed to replace the *tpx-1* orf with the *gfp* orf. PCR to confirm *tpx-1* KO was performed using gDNA extracted from parasites after drug selection. To examine the 5′ recombination event, primers tpx-1-5NR-integF and eGFP-R were used to amplify 1.2-kb DNA fragments. To confirm the 3′ recombination event, primers eGFP-F and tpx-1-3NR-integR were used to amplify 1.2-kb DNA fragments. To examine the existence of *tpx-1* orf, primers tpx-1-F and tpx-3NR-integR were used, by which 1.6-kb DNA fragments would be amplified from wild-type parasites.

### Introduction of a point mutation into B. bovis *tpx-1*.

The catalytic active site of B. bovis *tpx-1*, the peroxidatic Cys, was changed to Ser by transfecting parasites with the BbU6-Cas9-hDHFR-tpx-1-mutant plasmid. Transgenic parasites were cloned by limiting dilution, and the *tpx-1* orf was PCR amplified and sequenced to confirm the replacement mutation.

### Indirect immunofluorescence antibody test (IFAT).

Thin blood smears from cultured parasites were prepared and fixed as previously described (29). Blocking was done with 10% normal goat serum (Invitrogen)–PBS at 37°C for 30 min. To confirm the expression of Cas9, blood smears were immunostained with mouse anti-FLAG monoclonal antibody (M20008; Abmart, Shanghai, China) at 1:100 dilutions in PBS with 0.05% Tween 20 (T-PBS) and incubated at 37°C for 60 min. To evaluate myc epitope tagging of SBP3, the slides were immunostained with mouse anti-myc monoclonal antibody (9B11; Cell Signaling Technology, Danvers, MA, USA) at a 1:500 dilution. After 3 washes with T-PBS, the blood smears were incubated with Alexa Fluor 488-conjugated secondary goat anti-mouse IgG antibody (Invitrogen) (1:500 dilutions) at 37°C for 30 min. The smears were washed again 3 times with T-PBS and incubated with 1 μg/ml of Hoechst 33342 solution (Dojindo, Kumamoto, Japan) at 37°C for 20 min. After 3 washes with T-PBS, the smears were examined under a Nikon A1R confocal laser scanning microscope (Nikon, Tokyo, Japan).

### Southern blot analysis.

Genomic DNA was extracted from WT parasites and two *tpx-1* KO clones. Ten micrograms of DNA was digested overnight with 100 units of HindIII, AatII, or BamHI and SalI. The DNA was separated by agarose gel electrophoresis and then transferred onto HyBond N+ membranes (GE Healthcare, Buckinghamshire, United Kingdom). Two probes were used, one corresponding to the complete *gfp* orf and the other to a 0.5-kb length of the *cas9* orf, and both were labeled and hybridized by the use of an AlkPhos Direct kit (GE Healthcare). Chemiluminescent signal was developed with CDP-star detection reagent (GE Healthcare) and detected with a multipurpose charge-coupled-device (CCD) camera system (LAS-4000 mini EPUV; Fujifilm, Japan).

### Evaluation of parasite sensitivity to reactive nitrogen species (RNS).

B. bovis WT parasites or *tpx-1* KO or *tpx-1* mutant clones were cultured in the absence or presence of 10 μM sodium nitroprusside (SNP; Sigma) as a NO donor (30). Parasites were cultured in triplicate in 10% hematocrit with 0.1% as the initial parasitemia level for 4 days, and the culture medium was changed daily. Parasitemia was calculated by examining at least 10,000 RBCs on thin smears prepared on day 4. The growth rate of each transgenic parasite line was compared with that of the WT parasites and other transgenic lines by one-way analysis of variance (ANOVA) followed by Tukey’s multiple-comparison test, and growth differences were considered significant if the *P* value was below 0.05.
