# Supplementary figures and images for: Genome Editing of Babesia bovis Using the CRISPR/Cas9 System
Source: mSphere. 2019 Jun 12;4(3):e00109-19. doi: 10.1128/mSphere.00109-19 (PMC6563353; doi:10.1128/mSphere.00109-19)

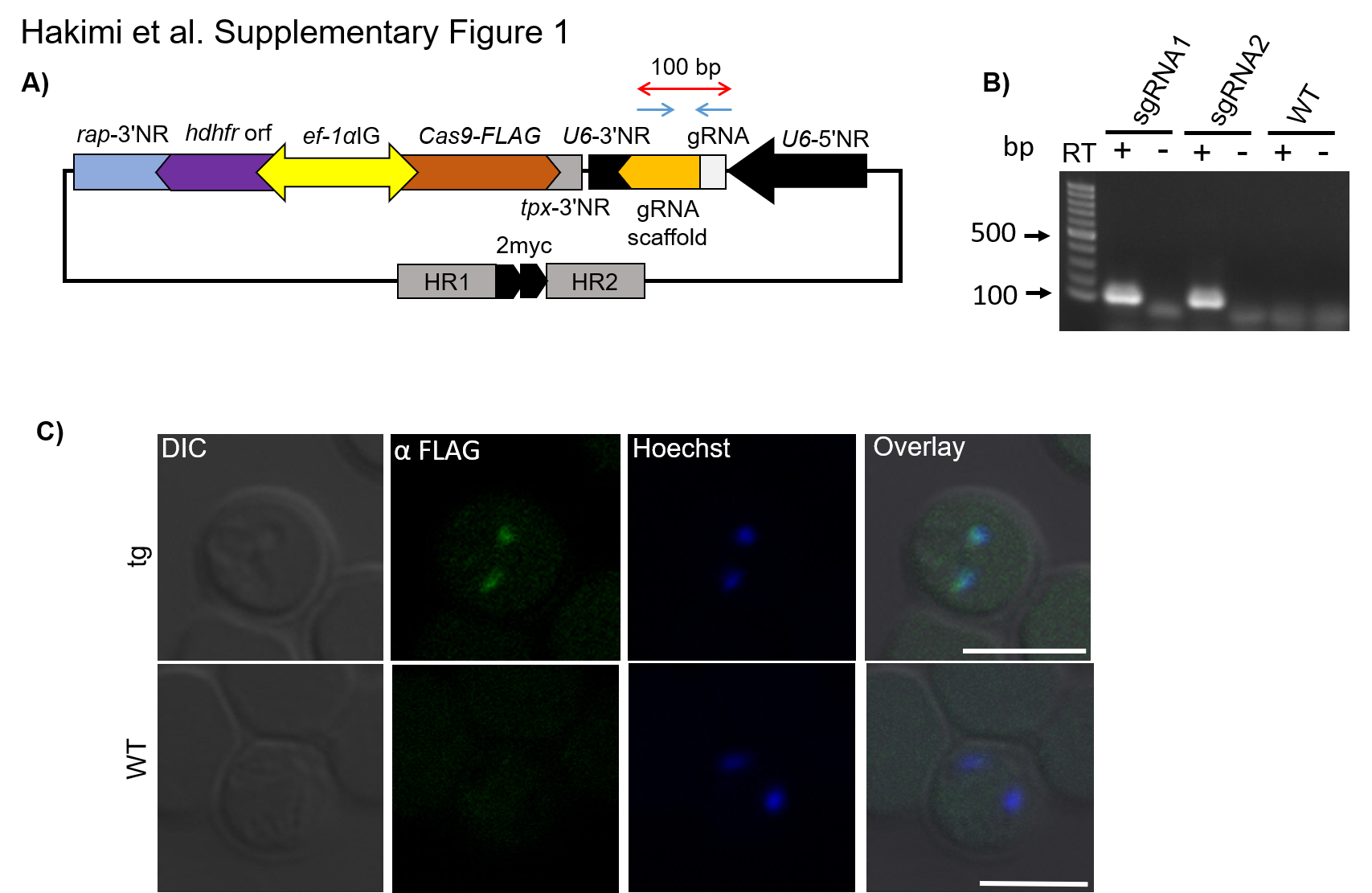

Supplement: FIG S1 [file mSphere.00109-19-sf001.tif]

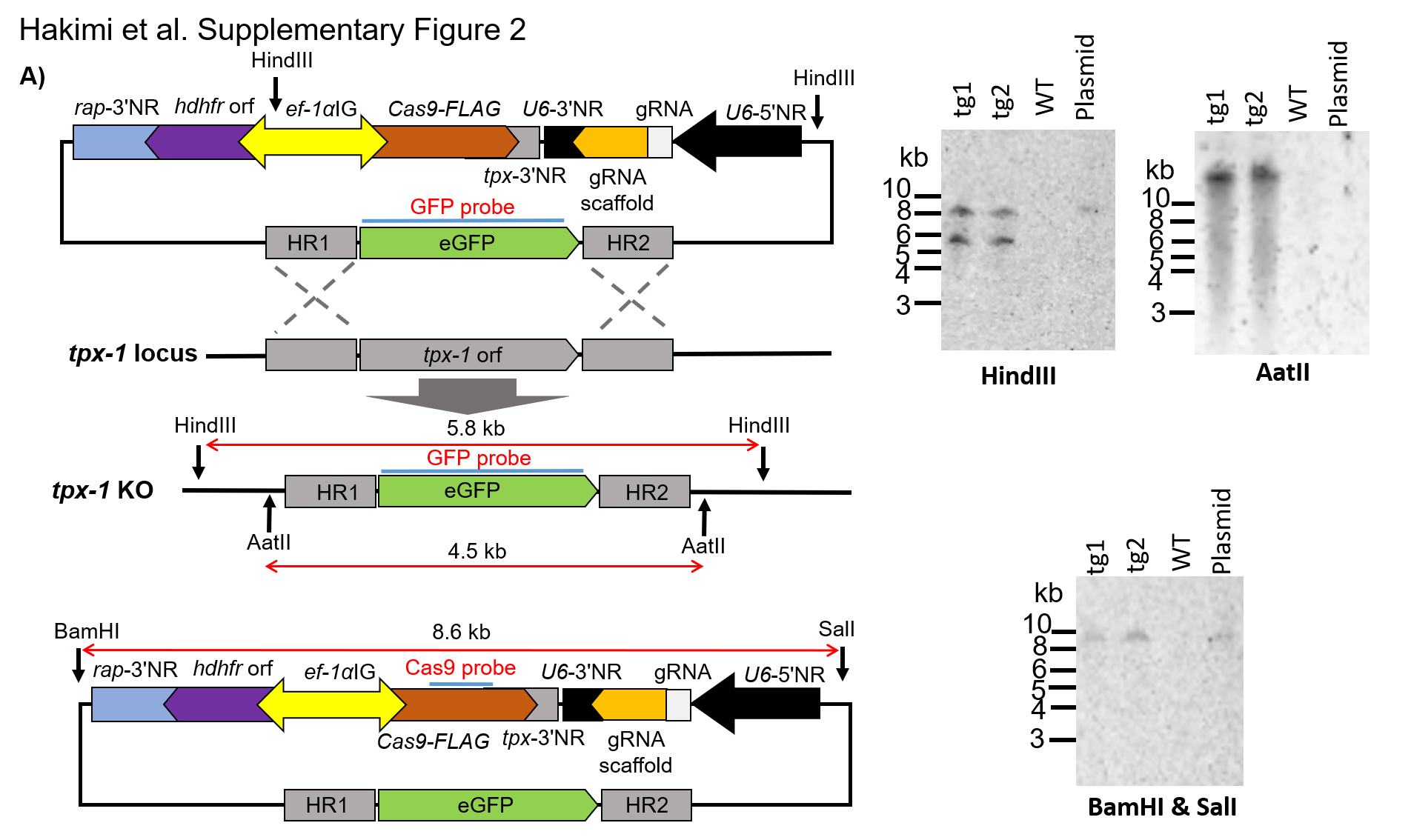

Supplement: FIG S2 [file mSphere.00109-19-sf002.tif]
